# Use of personal protective equipment among nurses in the pre- and post-COVID-19 vaccination periods*

**DOI:** 10.1590/1518-8345.7510.4731

**Published:** 2025-11-10

**Authors:** Wynne Pereira de Souza Nogueira, Elucir Gir, Mayra Gonçalves Menegueti, Maria Eliane Moreira Freire, Bárbara Iansã de Lima Barroso, Ana Cristina de Oliveira e Silva

**Affiliations:** 1 Universidade Federal da Paraíba, João Pessoa, PB, Brazil. Universidade Federal da Paraíba PB João Pessoa Brazil; 2 Universidade de São Paulo, Escola de Enfermagem de Ribeirão Preto, PAHO/WHO Collaborating Centre for Nursing Research Development, Ribeirão Preto, SP, Brazil. Universidade de São Paulo Escola de Enfermagem de Ribeirão Preto PAHO/WHO Collaborating Centre for Nursing Research Development SP Ribeirão Preto Brazil; 3 Universidade Federal de São Paulo, Departamento de Medicina Preventiva, São Paulo, SP, Brazil. Universidade Federal de São Paulo Departamento de Medicina Preventiva SP São Paulo Brazil

**Keywords:** Personal Protective Equipment, Nurses, COVID-19, Pandemics, Personal Protection, Disease Prevention

## Abstract

compare the use of personal protective equipment among nurses in the pre- and post-COVID-19 vaccination periods in the state of Paraíba, Brazil.

cross-sectional, analytical study with nurses from the state of Paraíba, Brazil. Data collection in two periods (pre- and post-COVID-19 vaccination), online, using a survey instrument. Descriptive and comparative analyses. Comparative analysis of variables for combined use of personal protective equipment in direct care and aerosol-generating procedures for people with suspected or diagnosed COVID-19 using the Z-test for proportions.

participation of 579 nurses in period 1 and 734 nurses in period 2. Gloves, face shield, N95 mask, surgical cap, and gown were the pieces of equipment most used by nurses in the care of people with suspected and/or diagnosed COVID-19. The combined use of personal protective equipment was higher in the pre-vaccination period compared with post-vaccination period.

combined use of personal protective equipment by nurses decreased after the introduction of the COVID-19 vaccine. Thus, it is necessary to implement educational strategies and trainings on the proper use of these pieces of equipment.

## Highlights

**(1)** Study with nurses in the pre- and post-COVID-19 vaccination periods. **(2)** Gloves, N95 mask, surgical cap, gown and face shield were the most used PPE. **(3)** Combined use of PPE was lower in the post-COVID-19 vaccination period.

## Introduction

The COVID-19 pandemic — caused by the viral etiological agent SARS-CoV-2 — was a global public health emergency that caused several economic and social impacts due to the easy transmissibility, high number of cases, of deaths, and overburdened health care systems. It is observed that, in the various COVID-19 pandemic contexts, health care professionals, especially nurses, played an indispensable role in controlling the pandemic, including the emphasis on contagion prevention measures, vaccination initiatives, support in tracing cases and contacts, and treatment of infected people^(1-2)^.

In the initial period of the COVID-19 pandemic, nurses dealt with increased working hours, complex clinical management of infected patients, precarious working conditions, lack of inputs and personal protective equipment (PPE), in addition to the higher risk of becoming ill from infection, due to higher exposure to the virus^(3)^. A systematic review study showed that nurses were the health care professionals most frequently infected with SARS-CoV-2^(4)^.

Thus, proper use of PPE has fundamental relevance for the protection of health care professionals, especially nurses, in the care of people diagnosed with the infection^(5)^. PPE refers to pieces of individual equipment aimed at preventing or minimizing worker exposure to different risks. In health care, such equipment is used to prevent and/or reduce the risks of transmission of and contamination by pathogens^(6)^.

As for COVID-19, the combined use of disposable gowns, gloves, face shields, protective eyewear, head covers/surgical caps, surgical masks and/or filtering facepiece respirators (PFF2/N95) was recommended and defined by protocols for health professionals working in the care of people with suspected and/or diagnosed infection^(7)^. In direct care of people with suspected or diagnosed COVID-19, such as care services, guidance and other care actions, health care professionals need to wear: head covers or surgical caps, surgical masks or N95/PFF2 respirators, gloves, waterproof or fabric gowns, coveralls or hood, and face shield, or protective eyewear. For aerosol-generating procedures, such as aspiration and nebulization, in the care of people under invasive and non-invasive mechanical ventilation, tracheostomized patients wear: head cover or surgical caps, gloves, N95/PFF2 masks or equivalent, waterproof gowns or coveralls, or hood and face shield, or protective eyewear^(7-9)^.

In the early pandemic phases, with unawareness of the disease, lack of effective treatment and lack of vaccine against COVID-19, the use of PPE and non-pharmacological measures were the main measures to prevent infection. Thus, due to the growing need for PPE, there was shortage and inadequate distribution of PPE worldwide^(10)^. In Brazil, a study showed that health care professionals did not receive sufficient training on the recommended and appropriate use of PPE in the care of people with COVID-19, which increased their propensity to become ill^(11)^.

In Brazil, a survey carried out in 2020, with 2,138 nurses, showed that 35% did not receive the necessary PPE in the first pandemic phase and 51.1% did not receive a specific course and/or training for use^(12)^. Moreover, a systematic review showed a prevalence of 8.1% of SARS-CoV-2 among nurses worldwide, with the Americas region presenting 8.4%^(13)^. Furthermore, all Brazilian regions were affected by the shortage of PPE^(11)^.

With the advent of COVID-19 vaccination, which represented a historic milestone in world science, there was a significant drop in the number of cases, deaths and hospitalization due to the disease. In Brazil, the first dose of the vaccine was administered on January 17, 2021, and, after more than 190 million doses were applied, the country saw the number of cases and deaths related to the infection drop by more than 50% by the end of 2021^(14)^.

However, due to the different dynamics of infection, vaccination rates, emergence of viral variants, use of non-pharmacological measures and planning of health care services, the use of adequate PPE remains an essential factor for the protection of health care professionals, especially nurses^(15)^, against COVID-19.

A study conducted in Birjand, Iran, showed that the low seroprevalence of SARS-CoV-2 among health care professionals can be explained by the good adherence to prevention and control measures against the infection and the appropriate use of PPE among professionals^(15)^. In addition, a systematic review showed that wearing PPE provided significant protection against COVID-19 infection compared with not wearing adequate PPE^(16)^.

During the COVID-19 pandemic, studies related to PPE for health care professionals as a means of protection against the disease were reported, but the vast majority of available evidence were focused on equipment the availability and accessibility^(17-18)^, safety and efficacy^(19)^, and the effects of prolonged use^(20)^.

Thus, due to the dynamics of infection, vaccination and the limited research on use of the PPE set in the pre- and post-COVID-19 vaccination periods among nurses — which results in lack of knowledge about the use of PPE recommended by government agencies in different periods of the pandemic —, this study aims to compare the use of PPE among nurses in the pre- and post-COVID-19 vaccination periods in the state of Paraíba, Brazil.

## Method

### Study design

This cross-sectional and analytical study followed the tool Strengthening the Reporting of Observational Studies in Epidemiology (STROBE) checklist^(21)^.

### Study location and period

This study was conducted in the state of Paraíba, located in the Northeast region of Brazil. The research was divided into two distinct periods: the first, from October 1 to December 31, 2020; and the second, from November 1, 2022 to May 1, 2023. These periods correspond to the pre- and post-COVID-19 vaccination periods, respectively.

With an estimated population of 3,974,687 inhabitants, the first period of the research presented an annual incidence of 1,228.93 cases per 100,000 inhabitants. The lethality rate was 1.59%, while the mortality rate was 19.51 deaths per 100,000 inhabitants, totaling 788 recorded deaths.

In the second phase of the research, the lethality rate was 0.27%, totaling 139 recorded deaths. No specific data on mortality rate during this period was found.

### Population

Nurses from the state of Paraíba who worked in public, private and/or philanthropic services.

### Inclusion criteria

Nurses who provided care to people in public, private and/or philanthropic services during the COVID-19 pandemic and who had access to the internet were considered eligible. Those who worked for less than six months after the onset of the COVID-19 pandemic were excluded.

### Sample definition

For the composition of the first sample, 13,581 nurses from Paraíba were considered; and for the second moment, 16,624, corresponding to the total number of nurses from Paraíba in 2020 and 2022^(22)^. For sample calculation, in both periods a 95% confidence interval, a desirable 5% margin of error and a 50% estimated frequency were assumed. A sample of 375 and 377 nurses was then determined for the first and second periods, respectively.

### Data collection instrument and study variables

The data collection instrument of the first moment of the research was divided into sections with multiple choice questions. The first section included data on sociodemographic information (biological sex, self-declared skin-color/race, marital status and education) and occupational information (sector of activity, type of institution in which they work, provided care in a field hospital for COVID-19, time of experience in the function); the second section included issues related to professional activity and the context of COVID-19 (provided care in a field hospital for COVID-19, diagnosis of COVID-19, received training or course on infection); and the third section included data on the availability and use of PPE (provision by the work institution of sufficient and quality PPE for use, which PPE were used in care and in aerosol-generating procedures for people with suspected or diagnosed COVID-19). The second-phase data collection instrument was similar, there was only the addition of one question related to the vaccination status (received a vaccine against COVID-19).

The data collection instrument was submitted to 15 evaluators for validation purposes. These experts filled out a form that contained items for general assessment of the data collection instrument (adequacy and applicability), of coherence and adequacy to the research objectives, as well as items to assess the scientific accuracy and content of the instrument and of the language assessment items (adequacy, clarity, objectivity). The Content Validity Index (CVI) was used and presented values above 0.85, considered valid.

The combined use of PPE in direct care and in procedures that generate aerosols in the care of people with suspected or diagnosed COVID-19 (surgical cap, gloves, N95 masks, gowns and face shields) were considered as dependent variables in both moments of the research.

### Data collection

The nurses from Paraíba were invited and recruited online, using the respondent driven sampling (RDS) method^(23)^ adapted to the virtual environment. In this method, the participating nurse recruited other people who met the same inclusion criteria for participation in the study.

It should be noted that, for better operationalization of online data collection, initially researchers were invited to act at this stage of the research as recruiters/collectors of participants who met the research criteria.

The implementation of the RDS began with the research coordinators, who sent the questionnaire to their professional peers, graduate students and former undergraduate students. The first participants, called “seeds,” received two social counterpart coupons, which were distributed to two other people, continuing the chain recruitment process. Importantly, participants were given no financial incentive to participate in the survey or to recruit new participants. This limitation was due to the lack of sufficient financial resources to cover all costs involved in the RDS sampling process.

The team of collectors was composed of undergraduate students, graduate students and health care professionals. Before recruitment, the team in charge carried out a previous training, via the Google Meet platform, presenting the research proposals and objectives, the eligibility criteria, the participant recruitment method and the data collection instrument, so they could clarify any doubts about the completion and the stage of the research.

Then, nurses who met the inclusion criteria were invited to participate in the research through invitations sent by social media (WhatsApp, Instagram, Facebook, email, LinkedIn). After acceptance of participation, we sent a link with information about the research and its confidentiality. Nurses, after clicking on the research link, were directed to the SurveyMonkey^®^ platform (first moment) and Research Electronic Data Capture (REDCap^®^) (second moment), which allowed a single submission of the form by internet protocol (IP).

On the first page of the link, the participant had access to the Informed Consent Form (ICF) in two copies, through its availability for download. After reading it and confirming participation, through the option “I have read and agree to participate in this research”, the participant was directed to the data collection instrument. In case of non-acceptance, they were directed to a closing page with a thanks message. Completing the questionnaire took an average of five to seven minutes and the recruiter in charge waited for the participant’s online feedback with confirmation of the completion of the survey and to answer any questions that might arise when completing the questionnaire.

### Data processing and analysis

Data were exported to a Microsoft Excel 2019 spreadsheet and subsequently imported and analyzed in the statistical software R, version 4.0.4, and in the Statistical Package for the Social Sciences (SPSS), version 20. Descriptive analysis was performed by means of distribution of absolute and relative frequencies for sample characterization.

Descriptive analyses were performed with absolute and relative frequencies. For comparative analyses of variables for combined use of PPE in direct care and aerosol-generating procedures for people with suspected or diagnosed COVID-19, we used the Z-test for proportions^(24)^.

### Ethical aspects

The research followed the rules established by Resolution No. 466/2012 of the National Health Council and the guidelines for procedures in research at any stage in a virtual environment, published and communicated by the National Research Ethics Committee (CONEP), on March 3, 2021, Circular Letter No. 02/2021^(25)^.

This study is linked to two studies, which were approved by the Research Ethics Committee (REC) of the responsible institution, under opinion No. 4.258.366/2020 (dated September 4, 2020) and No. 5.542.659/2021 (dated July 25, 2022), respectively.

## Results

In the first moment, 579 nurses from the state of Paraíba participated in the study. The majority were female (assigned at birth), 519 (89.6%); married or in stable union, 316 (54.6%); self-declared mixed-race, 291 (50.3%); with graduate education, 424 (73.2%). Regarding the nature of the work institution and the sector, most work in public institutions, 549 (94.8%); and in Basic Health Unit (BHU), 194 (33.5%), respectively (Table 1).

**Table 1-:** Sociodemographic, occupational and COVID-19-related characteristics of Paraíba nurses (n = 579). Paraíba, Brazil, 2020

**Characteristics**	**n**	**%**
**Sex**		
Female	519	89.6
Male	60	10.4
**Skin color/race**		
White	257	44.4
Mixed-race	291	50.3
Black	25	4.3
Yellow	6	1
**Marital status**		
Married/Stable union	316	54.6
Single/Divorced	259	44.7
Widowed	4	0.7
**Education**		
Higher Education	155	26.8
Post-graduate degree	424	73.2
Sector of activity*		
Outpatient clinic	39	6.7
ICU ^†^	124	21.4
Infirmary	129	22.3
Surgical center	41	7.1
Emergency care unit	59	10.2
Emergency Unit	103	17.8
Basic Health Unit	194	33.5
Private practice	3	0.5
Other	71	12.3
**Works in a public institution**		
Yes	549	94.8
No	30	5.2
**Works in a private institution**		
Yes	49	8.5
No	530	91.5
**Works in a philanthropic institution**		
Yes	6	1
No	573	99
**Provided care in a field hospital**		
Yes	157	27.1
No	422	72.9
**Diagnosed with COVID-19**		
Yes	174	30.1
No	405	69.9
**Received a course or training on COVID-19**		
Yes	387	66.8
No	192	33.2

*More than one response option; ^†^ICU = Intensive Care Unit

In the second period, 734 nurses from Paraíba participated in the study. The majority was female (assigned at birth), 642 (87.5%); married or in a stable union, 372 (50.7%); of self-declared mixed-race, 388 (52.9%); with specialization or residency, 440 (59.9%). As for the nature of the institution where they work, the majority is from a public institution, 622 (84.5%); and the work sector is the infirmary, 236 (32.2%) (Table 2).

**Table 2-:** Sociodemographic, occupational and COVID-19-related characteristics of Paraíba nurses (n = 734). Paraíba, Brazil, 2022-2023

**Variables**	**n**	**%**
**Sex**		
Female	642	87.5
Male	92	12.5
**Skin color/race**		
White	284	38.7
Mixed-race	388	52.9
Black	52	7.1
Yellow	8	1.1
Indigenous	2	0.3
**Marital status**		
Married/ stable union	372	50.7
Single/Divorced	358	48.8
Widowed	4	0.5
**Education**		
Higher Education	193	26.3
Post-graduate degree	541	73.7
Sector of activity*		
Outpatient clinic	77	10.5
ICU ^†^	159	21.6
Infirmary	236	32.2
Surgical center	71	9.7
Emergency care unit	127	17.3
Emergency Unit	149	20.3
Basic Health Unit	174	23.7
Private practice	20	2.7
Other	142	19.3
**Works in a public institution**		
Yes	622	84.5
No	112	15.5
**Works in a private institution**		
Yes	176	24
No	558	76
**Works in a philanthropic institution**		
Yes	21	2.9
No	713	97.1
**Diagnosed with COVID-19**		
Yes	522	71.1
No	212	28.9
**Vaccinated against COVID-19**		
Yes	656	89.4
No	78	10.6
**Received a course or training on COVID-19 in 2022**		
Yes	65	8.9
No	669	91.1

*More than one response option; ^†^ICU = Intensive Care Unit

Table 3 shows the frequency of use of PPE in direct care and aerosol-generating procedures for people with suspected or diagnosed COVID-19 in 2020. It is observed that, in direct care, the most used PPE were gloves, followed by face shield, surgical cap, N95 mask and waterproof gown. In aerosol-generating procedures, the most used PPE was the N95 mask, followed by the face shield, gloves, surgical cap and waterproof gown.

Table 4 shows the frequency of use of PPE in direct care and aerosol-generating procedures for people with suspected or diagnosed COVID-19 in 2022-2023. It is observed that the most used PPE were gloves, followed by the N95 mask, surgical cap, waterproof gown and face shield. In aerosol-generating procedures, the most used PPE was the N95 mask, followed by gloves, surgical cap, waterproof gown and face shield*.*

**Table 3-:** Distribution of PPE used by nurses in 2020 (n = 579). Paraíba, Brazil, 2020

PPE*	Direct care for people with suspected or diagnosed COVID-19 N (%)	Aerosol-generating procedures for people with suspected or diagnosed COVID-19 N (%)
**Gloves**		
Yes	525 (90.7)	453 (78.2)
No	54 (9.3)	126 (21.8)
**Face shield**		
Yes	508 (87.7)	479 (82.7)
No	71 (12.3)	100 (17.3)
**Surgical cap**		
Yes	503 (86.9)	444 (76.7)
No	76 (13.1)	135 (23.3)
**N95 mask**		
Yes	477 (82.4)	491 (84.8)
No	102 (17.6)	88 (15.2)
**Waterproof gown**		
Yes	377 (65.1)	359 (62)
No	202 (34.9)	220 (38)

*PPE = Personal Protective Equipment

**Table 4-:** Distribution of PPE used by nurses in the period of 2022-2023 (n = 734). Paraíba, Brazil, 2022-2023

PPE*	Direct care for people with suspected or diagnosed COVID-19 N (%)	Aerosol-generating procedures for people with suspected or diagnosed COVID-19 N (%)
**Gloves**		
Yes	578 (78.7)	531 (72.3)
No	156 (21.3)	203 (27.7)
**Face shield**		
Yes	368 (50.1)	399 (54.4)
No	366 (49.9)	335 (45.6)
**Surgical cap**		
Yes	512 (69.8)	457 (62.3)
No	222 (30.2)	277 (37.7)
**N95 mask**		
Yes	544 (74.1)	581 (79.2)
No	190 (25.9)	153 (20.8)
**Waterproof gown**		
Yes	431 (58.7)	424 (57.8)
No	303 (41.3)	310 (42.2)

*PPE = Personal Protective Equipment

As for the comparative results, Table 5 shows that there were statistically significant differences between the pre- and post-vaccination periods in the use of the PPE set in direct care and aerosol-generating procedures for people with suspected and/or diagnosed COVID-19. It is observed that, in 2020, the percentage of combined use of PPE in direct care for people with suspected and/or diagnosed COVID-19 by Paraíba nurses was higher (50.6%) compared with the use in 2022-2023 (34.5%) (p<0.01). Regarding the combined use of PPE in aerosol-generating procedures, it is also observed that the percentage of use was higher in 2020 (49.7%) compared with the use in 2022-2023 (32.1%) (p<0.01).

**Table 5-:** Comparative test of proportions of combined use of PPE for people with suspected and/or diagnosed COVID-19 among nurses from Paraíba. Paraíba, Brazil, 2020/2022-2023

Combined use of PPE* in direct care			
PPE*	**% in 2020**	**% in 2022-2023**	**P-value**
N95 + Gloves + Surgical cap + Gown + Face shield	50.6	34.5	< 0.01 ^†^
Combined use of PPE* in aerosol-generating procedures			
PPE*	**% in 2020**	**% in 2022-2023**	**P-value**
N95 + Gloves + Surgical cap + Gown + Face shield	49.7	32.1	**< 0.01** ^†^

*PPE = Personal protective equipment; ^†^ p<0.05

## Discussion

This study provides a comparative analysis of the combined use of PPE in direct care and aerosol-generating procedures for people with suspected and/or diagnosed COVID-19 among nurses in the pre- and post-COVID-19 vaccination period. Due to the limited research on the use of PPE among nurses at different times of the pandemic, studies carried out in similar periods and populations were used for comparative purposes. Thus, the research provides representative data for the Northeast region of Brazil.

Most respondents in the state of Paraíba are female assigned at birth, married or in a stable union and with a graduate degree. It is known that Nursing is the largest occupational group within the health care sector, with approximately more than 27 million professionals in the world^(26).^ The Nursing Research in Brazil showed that the majority are female (85.1%), 48.7% are married or in a stable union and 41.5% are mixed-race^(27)^, data that corroborate the findings of the study.

It is known that the use of PPE is essential for the protection of the health care professional, as well as the patient. The literature shows the importance and effectiveness of the use of PPE in the care of people with COVID-19, with the consequent reduction of contamination^(7,28)^. In the study, it is observed that the PPE most used by nurses in care, in both periods, were gloves, N95 mask, surgical cap, waterproof gown and face shield, that is, the Paraíba nurses used at least one of these PPE in the care of persons with suspected and/or diagnosed infection. These data corroborate the studies carried out with health care professionals from the state of Ceará^(29)^ and Pernambuco^(30)^.

Gloves, surgical cap and waterproof gown are PPE used routinely in patient health care that serve as barriers to the pathogen’s reach to body surfaces, such as hands, hair and body^(31-32)^. Regarding COVID-19, systematic reviews have demonstrated the effectiveness of PPE in preventing infection, noting that its protection becomes even more effective when combined with proper hand hygiene, both before and after the use of such equipment^(20,33)^.

Due to the transmission of SARS-CoV-2 by aerosols, the N95 mask and face shield have become essential PPE for nurses, especially in aerosol-generating procedures. Research shows that N95, due to its capacity to filter 95% of aerosolized particles, has a more effective protection against coronaviruses than surgical masks^(34)^. The face shield provides barrier protection for the facial area, which prevents droplets, fluids and/or aerosols from reaching the areas of the eyes, mouth or nose^(35)^. Notably, face shields do not replace the use of masks, but act as adjuvants in protection^(36)^.

In this study, it is observed that the N95 mask and the face shield were also PPE used more frequently. These data corroborate a study with 744 nurses from different continents^(37)^ and another study in the state of Pernambuco, Brazil^(30)^. Thus, it is observed that most nurses in the study used the main PPE, even if individually.

For higher protection of health care professionals against COVID-19, protocols and guidelines recommended the adoption of combined use of the main PPE: gowns, gloves, surgical caps, surgical and/or N95 mask, and eye protection (protective glasses/goggles or face shield)^(8,38)^. In 2020, the results showed that 50.6% of nurses used these PPE combined in direct care of people with suspected or diagnosed COVID-19. This is a higher result compared with a study in Egypt, in the first wave of COVID-19, in which 53.2% of health care professionals, including nurses, self-reported not using PPE^(39)^, and a lower result compared with a study in Germany, where adherence to combined use was 85%^(40)^.

As for the combined use of PPE in aerosol-generating procedures, 49.7% of nurses reported combined use. This result is lower compared with the result of a study with nurses from Saudi Arabia, where 100% used the recommended PPE in aerosol-generating procedures^(41)^. It is noted that Brazil, in the first pandemic moment, dealt with the shortage and inadequate distribution of PPE, and, in the country, there was a lack of courses and/or training on the infection and the correct gowning and de-gowning techniques, which may have impacted the low use and adherence to such equipment.

In 2022-2023, after the start of vaccination against COVID-19, 34.5% of Paraíba nurses used PPE combined in direct care. This is a lower result compared with a study conducted in Australia, with 2,197 health care professionals, which showed that 88.6% used PPE^(42)^ and in Denmark, with an adherence rate of 84.5%^(43)^. Regarding the combined use in aerosol-generating procedures for people with suspected or diagnosed COVID-19, 32.1% of nurses self-reported the use. This is a low percentage compared with a study conducted with middle and low-income countries, in which 49% and 67% of health care professionals reported the combined use of PPE, respectively^(17)^.

It is noteworthy that the world lives another situation after the mass vaccination of the population against COVID-19. In addition, the mandatory practice of wearing masks in health care services was suspended, except in some situations. Moreover, the use of PPE can be influenced by factors related to region, availability, training offered and fear of infection^(44)^. Such factors may have impacted the use of PPE at current times, requiring a critical-reflective approach to the adherence to standard precautions in health care settings.

In the comparative analysis, through statistically significant results, the study showed that in 2020 the percentage of use of PPE by nurses in direct care (50.6%) was higher compared with 2022-2023 (34.5%). There was also a similar result for the combined use in aerosol-generating procedures, in which 49.7% of nurses used PPE in 2020, and 32.1% in 2022-2023. There was a decrease of approximately 17% for both.

This is a concerning result, given that, despite the decreased number of cases and deaths, SARS-CoV-2 remains in circulation, and that, even after a serious global health crisis of a respiratory disease, the adoption of individual protection within health care services can still be neglected. A study conducted with Michigan health care professionals after the start of vaccination showed a significant increase in feelings of safety after the implementation of the vaccine and the mask use rate was higher in 2021^(45)^.

In addition, studies show that the credibility of the vaccine, the end of the public health emergency, stress of professionals due to prolonged use, decreased risk perception and beliefs, feelings and barriers are factors that can impact the preventive behavior and, consequently, the use of PPE^(46)^. It is also necessary to note that cultural beliefs that wearing PPE is no longer necessary and decreased perception of risk in relation to the virus may also be factors associated with the decreased use.

Therefore, the study provides important contributions as to the need for health care practices and policies aimed at the production and management of health and nursing care, especially in pandemic periods and related to the use of PPE. In addition, it emphasizes the need for training and education on the proper use of PPE.

Regarding the limitations of the study, it is noted a possible selection bias due to the higher representation of BHU professionals in the period before the vaccination and of infirmaries in the period after the vaccination. In addition, data collection occurred significantly after the implementation of the vaccines, which may have influenced the results.

## Conclusion

Gloves, face shield, surgical cap, N95 mask and waterproof gown were the PPE most frequently used by nurses from Paraíba in direct care and aerosol-generating procedures for people with suspected and/or diagnosed COVID-19. In comparing the use of PPE in the pre- and post-COVID-19 vaccination periods, it was observed that the combined use of PPE had a higher percentage in 2020, that is, in the pre-COVID-19 vaccination period, compared with the use in 2022-2023.

Therefore, it is still necessary to strengthen permanent education within health care institutions on the new scenarios and behavior of COVID-19, provide training on proper gowning, and provide updated guidelines on adherence to technical recommendations and/or protocols on the use of PPE as a respiratory protection barrier and the proper use of these pieces of equipment. Ensuring the nurses’ safety and well-being is essential for facing respiratory disease pandemics.

## Data Availability

Datasets related to this article will be available upon request to the corresponding author.
