# Supplementary material for: Use of personal protective equipment among nurses in the pre- and post-COVID-19 vaccination periods
Source: Rev Lat Am Enfermagem. 2025 Nov 10;33:e4731. doi: 10.1590/1518-8345.7510.4731 (PMC12610930; doi:10.1590/1518-8345.7510.4731)
Supplement: Translation

# Translation: Uso de equipamentos de proteção individual entre enfermeiros nos períodos pré e pós-vacinação contra a COVID-19*

**DOI:** 10.1590/1518-8345.7510.4732

**Authors:** Wynne Pereira de Souza Nogueira, Elucir Gir, Mayra Gonçalves Menegueti, Maria Eliane Moreira Freire, Bárbara Iansã de Lima Barroso, Ana Cristina de Oliveira e Silva

**Affiliations:** 1 Universidade Federal da Paraíba, João Pessoa, PB, Brasil. Universidade Federal da Paraíba PB João Pessoa Brasil; 2 Universidade de São Paulo, Escola de Enfermagem de Ribeirão Preto, Centro Colaborador da OPAS/OMS para o Desenvolvimento da Pesquisa em Enfermagem, Ribeirão Preto, SP, Brasil. Universidade de São Paulo Escola de Enfermagem de Ribeirão Preto Centro Colaborador da OPAS/OMS para o Desenvolvimento da Pesquisa em Enfermagem SP Ribeirão Preto Brasil; 3 Universidade Federal de São Paulo, Departamento de Medicina Preventiva, São Paulo, SP, Brasil. Universidade Federal de São Paulo Departamento de Medicina Preventiva SP São Paulo Brasil

**Keywords:** Equipamento de Proteção Individual, Enfermeiros, COVID-19, Pandemias, Proteção Pessoal, Prevenção de Doenças

## Abstract

comparar o uso de equipamentos de proteção individual entre enfermeiros nos períodos pré e pós-vacinação contra a COVID-19 no estado da Paraíba.

estudo transversal, analítico, realizado com enfermeiros do estado da Paraíba. Os dados foram coletados em dois períodos (pré e pós-vacinação contra a COVID-19), de forma *online*, por meio de um instrumento de pesquisa. Realizaram-se análises descritivas e comparativas. Para as análises comparativas das variáveis do uso em conjunto dos equipamentos de proteção individual na assistência direta e em procedimentos geradores de aerossóis às pessoas com suspeita ou diagnóstico de COVID-19, foi utilizado o Teste Z de proporções.

participaram 579 enfermeiros no período 1 e 734 enfermeiros no período 2. As luvas, o *face shield*, a máscara N95, o gorro e o avental foram os equipamentos mais utilizados pelos enfermeiros na assistência à pessoa suspeita e/ou diagnosticada com COVID-19. O uso em conjunto dos equipamentos de proteção individual foi maior no período de pré-vacinação quando comparado ao uso no pós-vacinação.

o uso do conjunto de equipamentos de proteção individual pelos enfermeiros diminuiu após o surgimento da vacina contra a COVID-19. Assim, faz-se necessária a implementação de estratégias educacionais e treinamentos sobre o uso adequado desses equipamentos.

## Destaques

**(1)** Estudo realizado com enfermeiros em período de pré e pós-vacinação contra a COVID-19. **(2)** Luvas, máscara N95, gorro, avental e *face shield* foram os EPI mais utilizados. **(3)** O uso do conjunto de EPI foi menor no período de pós-vacinação contra a COVID-19.

## Introdução

A pandemia da COVID-19, causada pelo agente etiológico viral SARS-CoV-2, foi uma emergência de saúde pública mundial que ocasionou diversos impactos econômicos e sociais por conta da fácil transmissibilidade, do alto número de casos, de óbitos e da superlotação dos serviços de saúde. É visto que, nos diversos cenários pandêmicos da COVID-19, os profissionais de saúde, sobretudo os enfermeiros, desempenharam um papel indispensável no controle da pandemia, desde a ênfase nas medidas de prevenção do contágio, ações de vacinação, apoio no rastreamento de casos e de contatos até o tratamento das pessoas contaminadas^(1-2)^.

No cenário inicial da COVID-19, enfermeiros lidaram com o aumento da jornada de trabalho, com a complexidade do manejo clínico de pacientes com a infecção, precarização das condições de trabalho, falta de insumos e de equipamentos de proteção individual (EPI), além do maior risco de adoecimento pela infecção, em decorrência da maior exposição ao vírus^(3)^. Um estudo de revisão sistemática mostrou que os enfermeiros foram os profissionais de saúde que apresentaram com maior frequência o diagnóstico de infecção pelo SARS-CoV-2^(4)^.

Assim, o uso adequado dos EPI é de relevância fundamental para a proteção dos profissionais de saúde, sobretudo os enfermeiros, no cuidado às pessoas diagnosticadas com a infecção^(5)^. Os EPI são equipamentos de uso individual que têm como finalidade a prevenção ou a minimização da exposição do trabalhador aos diferentes riscos. Na assistência à saúde, o uso desses equipamentos serve para prevenir e/ou diminuir os riscos de transmissão e de contaminação por patógenos^(6)^.

Na COVID-19, o uso em conjunto de aventais descartáveis, luvas, protetores faciais ou *face shield*, óculos de proteção, toucas/gorro, máscaras cirúrgicas e/ou respiradores de peças faciais filtrantes (PFF2/N95) foi recomendado e definido por protocolos para profissionais de saúde na assistência a pessoas suspeitas e/ou diagnosticadas com a infecção^(7)^. Na assistência direta à pessoa com suspeita ou com diagnóstico de COVID-19, como atendimentos, aconselhamento e demais ações assistenciais, os profissionais de saúde precisam utilizar: toucas ou gorros, máscaras cirúrgicas ou N95/PFF2, luvas, avental à prova d’água, ou avental de tecido, ou macacão, ou capote e *face shield*, ou óculos de proteção. Para procedimentos geradores de aerossóis, como aspiração e nebulizações, no cuidado de pessoas em uso de ventilação mecânica invasiva e não-invasiva, pacientes traqueostomizados utilizam: toucas ou gorros, luvas, máscaras N95/PFF2 ou equivalente, avental ou macacão à prova d’água, ou capote e *face shield*, ou óculos de proteção^(7-9)^.

Nas primeiras fases pandêmicas, o desconhecimento da doença, a falta de tratamento eficaz e a inexistência de vacina contra a COVID-19, o uso dos EPI acrescido das medidas não farmacológicas eram as principais medidas de prevenção da infecção. Dessa forma, em virtude da necessidade crescente desse material, houve a escassez e a distribuição inadequada de EPI em todo o mundo^(10)^. No Brasil, um estudo mostrou que profissionais de saúde não receberam treinamento suficiente sobre o uso recomendado e adequado dos EPI no atendimento às pessoas com COVID-19, o que aumentou a propensão destes ao adoecimento^(11)^.

No Brasil, uma pesquisa realizada em 2020, com 2.138 enfermeiros, mostrou que 35% não receberam EPI necessário na primeira fase pandêmica e 51,1% não receberam curso e/ou treinamento específico para uso^(12)^. Ademais, uma revisão sistemática mostrou uma prevalência de 8,1% de SARS-CoV-2 entre enfermeiros do mundo, com a região das Américas apresentando 8,4%^(13)^. Acrescenta-se ainda que todas as regiões brasileiras foram afetadas com a escassez de EPI^(11)^.

Com o advento da vacinação contra a COVID-19, marco histórico na ciência mundial, observou-se uma queda acentuada no número de casos, de óbitos e da necessidade de internação pela doença. No Brasil, a primeira dose do imunizante foi aplicada em 17 de janeiro de 2021, e após mais de 190 milhões de doses aplicadas, o país registrou uma queda de mais 50% no número de casos e de óbitos contra a infecção no final do ano de 2021^(14)^.

No entanto, em virtude das diferentes dinâmicas da infecção, dos índices de vacinação, do aparecimento de variantes virais, do uso de medidas não farmacológicas e do planejamento dos serviços de saúde, o uso de EPI adequados continua a ser uma condição essencial para a proteção dos profissionais de saúde, sobretudo dos enfermeiros^(15)^, contra a COVID-19.

Um estudo realizado em Birjand, Irã, mostrou que a baixa soroprevalência de SARS-CoV-2 entre os profissionais de saúde pode ser explicada pela boa adesão às medidas de prevenção e controle de infecções e pelo uso apropriado de EPI entre os profissionais^(15)^. Ainda, uma revisão sistemática mostrou que o uso de EPI conferiu proteção significativa contra infecção por COVID-19 em oposição a não usar EPI adequado^(16)^.

Durante a pandemia da COVID-19, estudos relacionados aos EPI para profissionais de saúde como forma de proteção contra a doença foram relatados, mas a grande maioria das evidências disponíveis focou a disponibilidade e acessibilidade aos equipamentos^(17-18)^, a segurança e eficácia^(19)^ e os efeitos do uso prolongado^(20)^.

Dessa forma, em virtude da dinâmica da infecção, da vacinação e da limitação de pesquisas sobre o uso do conjunto de EPI em períodos pré e pós-vacinação contra a COVID-19 entre os enfermeiros ‒ o que culmina no desconhecimento sobre a utilização dos EPI recomendados pelas agências governamentais em períodos distintos da pandemia –, este estudo tem como objetivo comparar o uso de EPI entre enfermeiros nos períodos pré e pós-vacinação contra a COVID-19 no estado da Paraíba.

## Método

### Delineamento do estudo

Estudo do tipo transversal e analítico guiado pela ferramenta *Strengthening the Reporting of Observational Studies in Epidemiology* (Strobe)^(21)^.

### Local e período

Este estudo foi realizado no estado da Paraíba, localizado na região Nordeste do Brasil. A pesquisa foi dividida em dois períodos distintos: o primeiro, compreendido entre 1º de outubro e 31 de dezembro de 2020; e o segundo, de 1º de novembro de 2022 a 1º de maio de 2023. Esses períodos correspondem à fase pré-vacinação e pós-vacinação contra a COVID-19, respectivamente.

Com uma população estimada em 3.974.687 de habitantes, o primeiro período da pesquisa apresentou uma incidência anual de 1.228,93 casos por 100 mil habitantes. A taxa de letalidade foi de 1,59%, enquanto a taxa de mortalidade foi de 19,51 óbitos por 100 mil habitantes, totalizando 788 óbitos registrados.

Na segunda fase da pesquisa, a taxa de letalidade foi de 0,27%, totalizando 139 óbitos registrados. Não foram encontrados dados específicos para a taxa de mortalidade durante esse período.

### População

Enfermeiros do estado da Paraíba que atuaram em serviços públicos, privados e/ou filantrópicos.

### Critérios de seleção

Foram considerados elegíveis enfermeiros que prestaram assistência às pessoas em serviços públicos, privados e/ou filantrópicos no período da pandemia da COVID-19 e que tinham acesso à internet. Excluíram-se aqueles que tiveram tempo de atuação inferior a seis meses após a declaração da pandemia da COVID-19.

### Definição da amostra

Para a composição da primeira amostra, foram considerados 13.581 enfermeiros paraibanos; e para o segundo momento, 16.624, correspondente ao número total de enfermeiros paraibanos em 2020 e em 2022^(22)^. Para o cálculo amostral, em ambos os períodos admitiu-se um intervalo de confiança de 95%, uma margem desejável de erro de 5% e uma frequência estimada de 50%. Determinou-se então uma amostra de 375 e 377 enfermeiros para o primeiro e o segundo período, respectivamente.

### Instrumento de coleta de dados e variáveis do estudo

O instrumento de coleta de dados do primeiro momento da pesquisa era dividido em seções com questões de múltipla escolha. A primeira seção contemplava dados sobre as informações sociodemográficas (sexo biológico, cor/raça autodeclarada, estado conjugal e escolaridade) e ocupacionais (setor de atuação, tipo de instituição em que trabalha, assistência em hospital de campanha para a COVID-19, tempo de experiência na função); a segunda, questões relacionadas à atuação profissional e ao contexto da COVID-19 (prestou assistência em hospital de campanha para a COVID-19, diagnóstico de COVID-19, recebeu capacitação ou curso sobre a infecção); e a terceira, sobre disponibilidade e uso de EPI (fornecimento pela instituição de trabalho de EPI suficiente e de qualidade para o uso, quais EPI utilizados na assistência e em procedimentos geradores de aerossóis às pessoas com suspeita ou diagnóstico de COVID-19). O instrumento da segunda fase de coleta de dados foi semelhante, apenas houve o acréscimo de questão relacionada à situação vacinal (recebeu vacina contra a COVID-19).

O instrumento de coleta de dados foi enviado para fins de validação a 15 avaliadores. Tais especialistas preencheram um formulário que continha itens de avaliação geral do instrumento de coleta de dados (adequação e aplicabilidade), de coerência e adequação aos objetivos da pesquisa, bem como itens para avaliar a precisão científica e o conteúdo do instrumento e dos itens de avaliação da linguagem (adequação, clareza, objetividade). O Índice de Validade de Conteúdo (IVC) foi utilizado e apresentou valores acima de 0,85, considerado válido.

Considerou-se como variáveis dependentes o uso em conjunto de EPI na assistência direta e na realização de procedimentos que geram aerossóis no atendimento às pessoas com suspeita ou diagnóstico de COVID-19 (gorro, luvas, máscaras N95, avental e protetor facial/*face shield*) em ambos os momentos da pesquisa.

### Coleta de dados

Os enfermeiros paraibanos foram convidados e recrutados de forma *online*, por meio do método *respondent-driven sampling* (RDS)^(23)^ adaptado para o ambiente virtual. Neste método, o enfermeiro participante recrutava outras pessoas que atendiam aos mesmos critérios de inclusão para a participação no estudo.

Convém ressaltar que, para uma melhor operacionalização da coleta de dados de forma *online*, inicialmente eram convidados pesquisadores para atuarem nesta fase da pesquisa como recrutadores/coletadores de participantes que atendiam aos critérios da pesquisa.

A implementação do RDS teve início com as coordenadoras da pesquisa, que enviaram o questionário para seus pares profissionais, orientandos de pós-graduação e ex-estudantes de graduação. Os primeiros participantes, denominados “sementes”, receberam dois cupons de contrapartida social, os quais foram distribuídos a outras duas pessoas, dando sequência ao processo de recrutamento de forma em cadeia. É importante ressaltar que os participantes não receberam nenhum incentivo financeiro para participar da pesquisa nem para recrutar novos participantes. Essa limitação ocorreu devido à falta de recursos financeiros suficientes para cobrir todos os custos envolvidos no processo de amostragem RDS.

A equipe de coletadores foi composta por estudantes de graduação, pós-graduação e profissionais de saúde. Antes do recrutamento, a equipe responsável realizou um treinamento prévio, por meio da plataforma *Google Meet*, no qual foram apresentadas as propostas e os objetivos da pesquisa, os critérios de elegibilidade, a forma de recrutamento dos participantes e o instrumento de coleta de dados, para que pudessem suprimir quaisquer dúvidas sobre o preenchimento e a etapa da pesquisa.

Então, os enfermeiros que atendiam aos critérios de inclusão foram convidados a participar da pesquisa por meio de convites enviados pelas mídias sociais (*WhatsApp*, *Instagram*, *Facebook*, *e-mail*, *LinkedIn*). Após o aceite em participar, enviava-se um link com informações sobre a pesquisa e sua confidencialidade. O enfermeiro, ao clicar no link da pesquisa, era direcionado à plataforma *SurveyMonkey*^®^ (primeiro momento) e *Research Electronic Data Capture* (*REDCap*^®^) (segundo momento), que permitia um único envio do formulário por protocolo de internet (IP).

Na primeira página do link, o participante tinha acesso ao Termo de Consentimento Livre e Esclarecido (TCLE) em duas vias, por meio da sua disponibilização para *download*. Após a leitura e confirmação da sua participação, por meio da opção “Li e concordo em participar da presente pesquisa”, o participante era direcionado para o instrumento de coleta de dados. Em caso de não aceitação, direcionava-se para uma página de encerramento com agradecimento. O preenchimento do questionário levava um tempo médio de cinco a sete minutos e o recrutador responsável aguardava o *feedbackonline* do participante com a confirmação da finalização da pesquisa e para sanar eventuais dúvidas que pudessem surgir ao preencher o questionário.

### Tratamento e análise dos dados

Os dados foram exportados para uma planilha do *Microsoft Excel* 2019 e, posteriormente, importados e analisados no *software* estatístico R, versão 4.0.4, e no *Statistical Package for the Social Sciences* (SPSS), versão 20. A análise descritiva foi realizada por meio de distribuição de frequências absolutas e relativas para a caracterização da amostra.

Foram realizadas análises descritivas com frequências absolutas e relativas. Para as análises comparativas das variáveis do uso em conjunto dos EPI na assistência direta e em procedimentos geradores de aerossóis a pessoas com suspeita ou com diagnóstico de COVID-19, foi utilizado o Teste Z de proporções^(24)^.

### Aspectos éticos

A pesquisa seguiu as normas estabelecidas pela Resolução nº 466/2012 do Conselho Nacional de Saúde e as orientações para procedimentos em pesquisas com qualquer etapa em ambiente virtual, publicadas e comunicadas pela Comissão Nacional de Ética e Pesquisa (CONEP), em 3 de março de 2021, por meio do Ofício Circular nº 02/2021^(25)^.

Este estudo está vinculado a duas pesquisas, as quais foram aprovadas pelo Comitê de Ética e Pesquisa (CEP) da instituição responsável, sob parecer de nº. 4.258.366/2020 (de 4 de setembro de 2020) e nº 5.542.659/2021 (de 25 de julho de 2022), respectivamente.

## Resultados

No primeiro momento participaram do estudo 579 enfermeiros do estado da Paraíba. A maioria do sexo feminino (designado ao nascimento), 519 (89,6%); casada ou em união estável, 316 (54,6%); raça autodeclarada parda, 291 (50,3%); com pós-graduação, 424 (73,2%). Quanto à natureza da instituição de trabalho e ao setor, a maioria trabalha em instituições públicas, 549 (94,8%); e em Unidade Básica de Saúde (UBS), 194 (33,5%), respectivamente (Tabela 1).

**Tabela 1-:** Características sociodemográficas, ocupacionais e relacionadas à COVID-19 dos enfermeiros da Paraíba (n = 579). Paraíba, Brasil, 2020

**Variáveis**	**n**	**%**
**Sexo**		
Feminino	519	89,6
Masculino	60	10,4
**Cor/Raça**		
Branca	257	44,4
Parda	291	50,3
Preta	25	4,3
Amarela	6	1
**Estado conjugal**		
Casado/União estável	316	54,6
Solteiro/Divorciado	259	44,7
Viúvo	4	0,7
**Escolaridade**		
Ensino Superior	155	26,8
Pós-Graduação	424	73,2
Setor de atuação*		
Ambulatório	39	6,7
UTI ^†^	124	21,4
Enfermaria	129	22,3
Centro cirúrgico	41	7,1
Unidade de Pronto Atendimento	59	10,2
Unidade de Emergência	103	17,8
Unidade Básica de Saúde	194	33,5
Consultório particular	3	0,5
Outro	71	12,3
**Trabalha em instituição pública**		
Sim	549	94,8
Não	30	5,2
**Trabalha em instituição privada**		
Sim	49	8,5
Não	530	91,5
**Trabalha em instituição filantrópica**		
Sim	6	1
Não	573	99
**Prestou assistência em hospital de campanha**		
Sim	157	27,1
Não	422	72,9
**Teve diagnóstico de COVID-19**		
Sim	174	30,1
Não	405	69,9
**Recebeu curso ou capacitação sobre a COVID-19**		
Sim	387	66,8
Não	192	33,2

*Mais de uma opção de resposta; ^†^UTI = Unidade de Terapia Intensiva

No segundo período participaram do estudo 734 enfermeiros paraibanos. A maioria do sexo feminino (designado ao nascimento), 642 (87,5%); casada ou em união estável, 372 (50,7%); da raça autodeclarada parda, 388 (52,9%); com especialização ou residência, 440 (59,9%). Quanto à natureza da instituição onde trabalha, a maioria é de instituição pública, 622 (84,5%); e o setor de trabalho são as enfermarias, 236 (32,2%) (Tabela 2).

**Tabela 2-:** Características sociodemográficas, ocupacionais e relacionadas à COVID-19 dos enfermeiros da Paraíba (n = 734). Paraíba, Brasil, 2022-2023

**Variáveis**	**n**	**%**
**Sexo**		
Feminino	642	87,5
Masculino	92	12,5
**Cor/Raça**		
Branca	284	38,7
Parda	388	52,9
Preta	52	7,1
Amarela	8	1,1
Indígena	2	0,3
**Estado conjugal**		
Casado/União estável	372	50,7
Solteiro/Divorciado	358	48,8
Viúvo	4	0,5
**Escolaridade**		
Ensino Superior	193	26,3
Pós-Graduação	541	73,7
Setor de atuação*		
Ambulatório	77	10,5
UTI ^†^	159	21,6
Enfermaria	236	32,2
Centro cirúrgico	71	9,7
Unidade de Pronto Atendimento	127	17,3
Unidade de Emergência	149	20,3
Unidade Básica de Saúde	174	23,7
Consultório particular	20	2,7
Outro	142	19,3
**Trabalha em instituição pública**		
Sim	622	84,5
Não	112	15,5
**Trabalha em instituição privada**		
Sim	176	24
Não	558	76
**Trabalha em instituição filantrópica**		
Sim	21	2,9
Não	713	97,1
**Teve diagnóstico de COVID-19**		
Sim	522	71,1
Não	212	28,9
**Vacinado contra a COVID-19**		
Sim	656	89,4
Não	78	10,6
**Recebeu curso ou capacitação sobre a COVID-19 em 2022**		
Sim	65	8,9
Não	669	91,1

*Mais de uma opção de resposta; ^†^UTI = Unidade de Terapia Intensiva

A Tabela 3 mostra a frequência de utilização dos EPI na assistência direta e em procedimentos geradores de aerossóis às pessoas com suspeita ou diagnóstico de COVID-19 no ano de 2020. Observa-se que, na assistência direta, os EPI mais utilizados foram as luvas, seguidas do *face shield* ou protetor facial, gorro, máscara N95 e avental impermeável. Já em procedimentos geradores de aerossóis, o EPI mais utilizado foi a máscara N95, seguida pelo *face shield* ou protetor facial, luvas, gorro e avental impermeável.

A Tabela 4 mostra a frequência de utilização dos EPI na assistência direta e em procedimentos geradores de aerossóis às pessoas com suspeita ou diagnóstico de COVID-19 no ano de 2022-2023. Observa-se que o EPI mais utilizado foram as luvas, seguido da máscara N95, gorro, avental impermeável e *face shield*. Já em procedimentos geradores de aerossóis, o mais utilizado foi a máscara N95, seguido por luvas, gorro, avental impermeável e *face shield.*

**Tabela 3-:** Distribuição dos EPI utilizados pelos enfermeiros no ano de 2020 (n = 579). Paraíba, Brasil, 2020

EPI*	Assistência direta a pessoas com suspeita ou diagnóstico de COVID-19 N (%)	Procedimentos geradores de aerossóis a pessoas com suspeita ou diagnóstico de COVID-19 N (%)
**Luvas**		
Sim	525 (90,7)	453 (78,2)
Não	54 (9,3)	126 (21,8)
***Face* *shield***		
Sim	508 (87,7)	479 (82,7)
Não	71 (12,3)	100 (17,3)
**Gorro**		
Sim	503 (86,9)	444 (76,7)
Não	76 (13,1)	135 (23,3)
**Máscara N95**		
Sim	477 (82,4)	491 (84,8)
Não	102 (17,6)	88 (15,2)
**Avental impermeável**		
Sim	377 (65,1)	359 (62)
Não	202 (34,9)	220 (38)

*EPI = Equipamentos de proteção individual

**Tabela 4-:** Distribuição dos EPI utilizados pelos enfermeiros no período de 2022-2023 (n = 734). Paraíba, Brasil, 2022-2023

EPI*	Assistência direta a pessoas com suspeita ou diagnóstico de COVID-19 N (%)	Procedimentos geradores de aerossóis a pessoas com suspeita ou diagnóstico de COVID-19 N (%)
**Luvas**		
Sim	578 (78,7)	531 (72,3)
Não	156 (21,3)	203 (27,7)
***Face shield***		
Sim	368 (50,1)	399 (54,4)
Não	366 (49,9)	335 (45,6)
**Gorro**		
Sim	512 (69,8)	457 (62,3)
Não	222 (30,2)	277 (37,7)
**Máscara N95**		
Sim	544 (74,1)	581 (79,2)
Não	190 (25,9)	153 (20,8)
**Avental impermeável**		
Sim	431 (58,7)	424 (57,8)
Não	303 (41,3)	310 (42,2)

*EPI = Equipamentos de proteção individual

Quanto aos resultados comparativos, observa-se na Tabela 5 que houve diferenças estatisticamente significativas entre os períodos de pré e pós-vacinação na utilização do conjunto de EPI na assistência direta e em procedimentos geradores de aerossóis às pessoas com suspeita e/ou diagnóstico de COVID-19. Observa-se que, no ano de 2020, o percentual do uso em conjunto dos EPI na assistência direta a pessoas com suspeita e/ou diagnóstico de COVID-19 pelos enfermeiros paraibanos foi maior (50,6%), quando comparado ao uso no ano de 2022-2023 (34,5%) (p<0,01). Em relação ao uso em conjunto dos EPI em procedimentos geradores de aerossóis, observa-se também que o percentual de uso foi maior em 2020 (49,7%), quando comparado ao uso em 2022-2023 (32,1%) (p<0,01).

**Tabela 5-:** Teste de comparação de proporções do uso em conjunto de EPI a pessoas com suspeita e/ou diagnóstico de COVID-19 entre enfermeiros paraibanos. Paraíba, Brasil, 2020/2022-2023

Uso em conjunto dos EPI* na assistência direta			
EPI*	**% em 2020**	**% em 2022-2023**	**P-valor**
N95 + Luvas + Gorro + Avental + *Face shield*	50,6	34,5	< 0,01 ^†^
Uso em conjunto dos EPI* em procedimentos geradores de aerossóis			
EPI*	**% em 2020**	**% em 2022-2023**	**P-valor**
N95 + Luvas + Gorro + Avental + *Face shield*	49,7	32,1	**< 0,01** ^†^

*EPI = Equipamentos de proteção individual; ^†^ p<0,05

## Discussão

Este estudo traz uma análise comparativa do uso em conjunto de EPI na assistência direta e em procedimentos geradores de aerossóis às pessoas suspeitas e/ou diagnosticadas com COVID-19 entre enfermeiros em período pré e pós-vacinação contra a infecção. Em virtude da limitação de pesquisas sobre o uso de EPI entre enfermeiros em momentos distintos da pandemia, foram utilizados estudos realizados em períodos e com populações semelhantes para fins comparativos. Assim, a pesquisa traz dados representativos para a região Nordeste do Brasil.

A maioria das pessoas respondentes, no estado da Paraíba, é do sexo feminino designado ao nascimento, casada ou em união estável e com pós-graduação. Sabe-se que a Enfermagem é o maior grupo ocupacional dentro do setor saúde, contendo, aproximadamente, mais de 27 milhões de profissionais no mundo^(26).^ A Pesquisa de Enfermagem no Brasil mostrou que a maioria é do sexo feminino (85,1%), 48,7% é casada ou em união estável e 41,5% é da raça parda^(27)^, dados que corroboram os achados do estudo.

Sabe-se que o uso de EPI é fundamental para a proteção do profissional de saúde, bem como do paciente. A literatura deixa evidente a importância e a eficácia do uso de EPI no cuidado à pessoa com COVID-19, com a consequente redução da contaminação^(7,28)^. No estudo, observa-se que os EPI mais utilizados pelos enfermeiros na assistência, em ambos os períodos, foram as luvas, a máscara N95, o gorro, o avental impermeável e o *face shield*, ou seja, os enfermeiros paraibanos utilizaram pelo menos um desses EPI no cuidado à pessoa suspeita e/ou diagnosticada com a infecção. Dados que corroboram os estudos realizados com profissionais de saúde do estado do Ceará^(29)^ e de Pernambuco^(30)^.

As luvas, o gorro e o avental impermeável são EPI utilizados como rotina na assistência à saúde ao paciente que serve como barreira do alcance do patógeno para as superfícies corporais, como mãos, cabelos e corpo^(31-32)^. Em relação à COVID-19, revisões sistemáticas demonstraram a eficácia dos EPI na prevenção da infecção, destacando que sua proteção se torna ainda mais eficaz quando combinada com a higienização adequada das mãos, tanto antes quanto após o uso dos equipamentos^(20,33)^.

Em decorrência da transmissão do SARS-CoV-2 por aerossóis, a máscara N95 e o *face shield* tornaram-se EPI essenciais para o enfermeiro, especialmente em procedimentos geradores de aerossóis. Pesquisas mostram que a N95, pela sua capacidade de filtração de 95% das partículas aerossolizadas, tem uma proteção mais eficaz contra os coronavírus do que as máscaras cirúrgicas^(34)^. Já o protetor facial (*face shield*) fornece proteção de barreira para a área facial, a qual impede que gotículas, fluidos e/ou aerossóis atinjam as áreas dos olhos, boca ou nariz^(35)^. É importante mencionar que os protetores faciais não substituem o uso de máscaras, e sim atuam como coadjuvantes na proteção^(36)^.

Neste estudo, observa-se que a máscara N95 e o *face shield* também foram EPI utilizados com maior frequência. Dados que corroboram uma pesquisa realizada com 744 enfermeiros de diferentes continentes^(37)^ e outra realizada no estado de Pernambuco, Brasil^(30)^. Assim, verifica-se que a maioria dos enfermeiros do estudo fez uso dos principais EPI, mesmo que de forma individual.

Para uma maior proteção do profissional de saúde contra a COVID-19, protocolos e diretrizes recomendaram a adoção do uso em conjunto dos principais EPI: aventais, luvas, gorros, máscara cirúrgica e/ou N95 e proteção para olhos (óculos de proteção ou *face shield*)^(8,38)^. No ano de 2020, os resultados mostraram que 50,6% dos enfermeiros fizeram uso em conjunto desses EPI na assistência direta às pessoas com suspeita ou diagnóstico de COVID-19. Resultado superior quando comparado a um estudo realizado no Egito, na primeira onda de COVID-19, em que 53,2% dos profissionais de saúde, incluindo enfermeiros, autorrelataram o não uso de EPI^(39)^ e tiveram resultado inferior quando comparado a um estudo realizado na Alemanha, onde a adesão ao uso em conjunto foi de 85%^(40)^.

Já o uso em conjunto dos EPI em procedimentos geradores de aerossóis, 49,7% dos enfermeiros referiram a utilização conjunta. Dado inferior quando comparado ao resultado de um estudo realizado com enfermeiros da Arábia Saudita, onde 100% desses utilizaram os EPI recomendados em procedimentos geradores de aerossóis^(41)^. Ressalta-se que o Brasil, no primeiro momento pandêmico, lidou com a escassez e a distribuição inadequada dos EPI, bem como, no país, houve a falta de cursos e/ou capacitação sobre a infecção e as técnicas corretas de paramentação e desparamentação, o que pode ter impactado o baixo uso e adesão aos equipamentos.

No ano de 2022-2023, após o início da vacinação contra COVID-19, 34,5% dos enfermeiros paraibanos fizeram uso em conjunto dos EPI na assistência direta. Resultado inferior quando comparado a um estudo realizado na Austrália, com 2.197 profissionais de saúde, que mostrou que 88,6% utilizaram EPI^(42)^ e na Dinamarca, com taxa de adesão de 84,5%^(43)^. Em relação ao uso em conjunto em procedimentos geradores de aerossóis às pessoas com suspeita ou diagnóstico de COVID-19, 32,1% dos enfermeiros autorrelataram o uso. Baixa porcentagem quando comparada a um estudo realizado com países de média e baixa renda, em que 49% e 67% dos profissionais de saúde relataram o uso em conjunto dos EPI, respectivamente^(17)^.

É mister abordar que o mundo vive outro cenário após a vacinação em massa da população contra a COVID-19. Ademais, a prática obrigatória do uso de máscaras em serviços de saúde foi suspensa, exceto em algumas situações. E ainda o uso de EPI pode ser influenciado por fatores regionais, de disponibilidade, treinamento ofertado e o medo da infecção^(44)^. Tais fatores podem ter impactado o uso de EPI em momentos atuais, exigindo-se um olhar crítico-reflexivo sobre a adesão às precauções padrão em ambientes de saúde.

Na análise comparativa, por meio de resultados estatisticamente significantes, o estudo mostrou que no ano de 2020 a porcentagem de uso de EPI pelos enfermeiros na assistência direta (50,6%) foi maior quando comparada ao ano de 2022-2023 (34,5%). Resultado também semelhante para o uso em conjunto em procedimentos geradores de aerossóis, em que 49,7% dos enfermeiros fizeram uso em 2020, e 32,1% em 2022-2023. Uma diminuição de aproximadamente 17% para ambos.

Resultado preocupante, uma vez que, apesar da diminuição do número de casos e de óbitos, o SARS-CoV-2 permanece em circulação, e que mesmo após uma grave crise sanitária global de uma doença respiratória, a adoção de proteção individual dentro dos serviços de saúde pode ainda ser negligenciada. Um estudo realizado com profissionais de saúde de Michigan, após o início da vacinação, mostrou um aumento significativo nos sentimentos de segurança após a implementação da vacina e a taxa do uso de máscaras foi maior em 2021^(45)^.

Ademais, estudos evidenciam que a segurança na vacina, o fim da emergência de saúde pública, o desgaste do profissional em decorrência do uso prolongado, a diminuição da percepção de risco e as crenças, os sentimentos e as barreiras são fatores que podem impactar o comportamento preventivo e, consequentemente, o uso de EPI^(46)^. É mister abordar também que as crenças culturais de que a utilização do EPI não se faz mais necessária e a diminuição da sua percepção de risco diante do vírus também podem ser fatores associados à diminuição do uso.

Logo, o estudo traz importantes contribuições sobre a necessidade de práticas e de políticas de saúde voltadas para a produção e gestão do cuidado em saúde e de enfermagem, especialmente em períodos pandêmicos e relacionados ao uso de EPI. Além disso, enfatiza a necessidade de treinamento e de educação sobre a utilização adequada dos EPI.

Quanto às limitações do estudo, ressalta-se um possível viés de seleção, decorrente da maior representatividade de profissionais das UBS no período anterior à vacinação e de enfermarias no período pós-vacinação. Além disso, a coleta de dados ocorreu em um momento significativamente posterior à implementação das vacinas, o que pode ter influenciado os resultados.

## Conclusão

As luvas, o *face shield* ou protetor facial, o gorro, a máscara N95 e o avental impermeável foram os EPI mais frequentemente utilizados pelos enfermeiros paraibanos na assistência direta e em procedimentos geradores de aerossóis para pessoas suspeitas e/ou diagnosticadas com COVID-19. Ao comparar o uso de EPI no período de pré e pós-vacinação da COVID-19, observou-se que o uso em conjunto de EPI foi percentualmente maior em 2020, ou seja, no período pré-vacinação contra a COVID-19, quando comparado ao uso nos anos de 2022-2023.

Portanto, ainda se faz necessário o fortalecimento da educação permanente dentro das instituições de saúde sobre os novos cenários e comportamento da COVID-19, a oferta de treinamentos sobre a paramentação adequada, assim como da atualização para a adesão às recomendações técnicas e/ou aos protocolos sobre o uso de EPI como barreira de proteção respiratória e do uso adequado desses equipamentos. A garantia de segurança e do bem-estar dos enfermeiros é essencial para o enfrentamento de pandemias de doenças respiratórias.

## Data Availability

Os conjuntos de dados relacionados a este artigo estarão disponíveis mediante solicitação ao autor correspondente.

# Translation: Uso de equipos de protección individual entre enfermeros en los períodos pre- y posvacunación contra COVID-19*

**DOI:** 10.1590/1518-8345.7510.4730

**Authors:** Wynne Pereira de Souza Nogueira, Elucir Gir, Mayra Gonçalves Menegueti, Maria Eliane Moreira Freire, Bárbara Iansã de Lima Barroso, Ana Cristina de Oliveira e Silva

**Affiliations:** 1 Universidade Federal da Paraíba, João Pessoa, PB, Brasil. Universidade Federal da Paraíba PB João Pessoa Brasil; 2 Universidade de São Paulo, Escola de Enfermagem de Ribeirão Preto, Centro Colaborador de la OPS/OMS para el Desarrollo de la Investigación en Enfermería, Ribeirão Preto, SP, Brasil. Universidade de São Paulo Escola de Enfermagem de Ribeirão Preto Centro Colaborador de la OPS/OMS para el Desarrollo de la Investigación en Enfermería SP Ribeirão Preto Brasil; 3 Universidade Federal de São Paulo, Departamento de Medicina Preventiva, São Paulo, SP, Brasil. Universidade Federal de São Paulo Departamento de Medicina Preventiva SP São Paulo Brasil

**Keywords:** Equipo de Protección Personal, Enfermeros, COVID-19, Pandemias, Protección Personal, Prevención de Enfermedades

## Abstract

comparar el uso de equipos de protección individual entre enfermeros en los períodos pre- y posvacunación contra COVID-19 en el estado de Paraíba.

estudio transversal, analítico, realizado con enfermeros del estado de Paraíba. Los datos se recopilaron en dos períodos (pre- y posvacunación contra COVID-19), en línea, utilizando un instrumento de investigación. Se realizaron análisis descriptivos y comparativos. Para análisis comparativos de las variables del uso combinado de equipos de protección individual en la asistencia directa y en procedimientos generadores de aerosoles en personas con sospecha o con diagnóstico de COVID-19, se utilizó la prueba Z de proporciones.

participaron 579 enfermeros en el período 1 y 734 enfermeros en el período 2. Los guantes, la pantalla facial, la mascarilla N95, el gorro y la bata fueron los equipos más utilizados por los enfermeros en la asistencia a personas con sospecha o con diagnóstico de COVID-19. El uso combinado de los equipos de protección individual fue mayor en el período prevacunación en comparación con el uso en el período posvacunación.

el uso de equipos de protección individual por parte de los enfermeros disminuyó tras la aparición de la vacuna contra COVID-19. Por lo tanto, es necesario implementar estrategias educativas y capacitación sobre el uso adecuado de estos equipos.

## Destacados

**(1)** Estudio realizado con enfermeros en el período pre- y posvacunación contra COVID-19. **(2)** Guantes, mascarilla N95, gorro, bata y protector facial fueron los EPI más utilizados. **(3)** El uso combinado de EPI fue menor en el periodo posvacunación contra COVID-19.

## Introducción

La pandemia de COVID-19, causada por el agente etiológico viral SARS-CoV-2, fue una emergencia de salud pública mundial que provocó diversos impactos económicos y sociales debido a su fácil transmisibilidad, al elevado número de casos, de muertes y a la sobresaturación de los servicios de salud. Se observa que, en los diversos escenarios pandémicos de COVID-19, los profesionales de salud, especialmente los enfermeros, desempeñaron un papel indispensable en el control de la pandemia enfatizando las medidas de prevención del contagio, realizando acciones de vacunación, apoyando el rastreo de casos y contactos e incluso tratando a las personas infectadas^(1-2)^.

En el escenario inicial de COVID-19, los enfermeros se enfrentaron al aumento de la jornada laboral, a la complejidad del manejo clínico de los pacientes con la infección, a las precarias condiciones de trabajo, a la falta de insumos y equipos de protección individual (EPI), además del mayor riesgo de enfermar por la infección, debido a la mayor exposición al virus^(3)^. Un estudio de revisión sistemática mostró que los enfermeros fueron los profesionales de salud a los que se les diagnosticó con mayor frecuencia infección por SARS-CoV-2^(4)^.

Por lo tanto, el uso adecuado de los EPI es de fundamental importancia para la protección de los profesionales de salud, especialmente los enfermeros, en la atención a personas diagnosticadas con la infección^(5)^. Los EPI son equipos de uso individual que tienen como objetivo prevenir o minimizar la exposición de los trabajadores a diferentes riesgos. En el ámbito sanitario, el uso de estos equipos sirve para prevenir o reducir los riesgos de transmisión y contaminación por patógenos^(6)^.

Durante la pandemia de COVID-19, el uso combinado de batas descartables, guantes, protectores o pantallas faciales, gafas protectoras, gorros o cofias, mascarillas quirúrgicas o respiradores con piezas faciales filtrantes (PFF2/N95) fue recomendado y establecido por protocolos para profesionales de salud en la asistencia a personas con sospecha o con diagnóstico de infección^(7)^. En la asistencia directa a personas con sospecha o diagnóstico de COVID-19, como atenciones, asesoramiento y otras acciones asistenciales, los profesionales de salud deben utilizar: gorros o cofias, mascarillas quirúrgicas o N95/PFF2, guantes, bata impermeable, o bata de tela, o mono, o chaqueta, y pantalla facial, o gafas protectoras. Para procedimientos generadores de aerosoles, como aspiración y nebulizaciones, en el cuidado de personas que utilizan ventilación mecánica invasiva y no invasiva, pacientes traqueostomizados usan: gorros o cofias, guantes, mascarillas N95/PFF2 o equivalente, bata o mono impermeable, o bata y pantalla facial, o gafas protectoras^(7-9)^.

En las primeras etapas pandémicas, debido al desconocimiento de la enfermedad, a la falta de un tratamiento efectivo y a la falta de una vacuna contra COVID-19, el uso de EPI combinado con medidas no farmacológicas fueron las principales medidas para prevenir la infección. Así, en virtud de la necesidad creciente de ese material, hubo escasez y una distribución inadecuada de EPI en todo el mundo^(10)^. En Brasil, un estudio mostró que los profesionales de salud no recibieron capacitación suficiente sobre el uso recomendado y adecuado de los EPI para atender a personas con COVID-19, lo que aumentó su propensión a enfermarse^(11)^.

En Brasil, una encuesta realizada en el 2020, con 2.138 enfermeros, mostró que el 35% no recibió los EPI necesarios en la primera etapa de la pandemia, y el 51,1% no recibió un curso o capacitación específica para su uso^(12)^. Además, una revisión sistemática mostró una prevalencia del 8,1% de SARS-CoV-2 entre enfermeros en el ámbito mundial, y la región de las Américas presentó un 8,4%^(13)^. Cabe añadir también que todas las regiones brasileñas se vieron afectadas por la escasez de EPI^(11)^.

Con el advenimiento de la vacunación contra COVID-19, marco histórico en la ciencia mundial, se observó una reducción acentuada en el número de casos, de muertes y en la necesidad de hospitalización debido a la enfermedad. En Brasil, la primera dosis del inmunizante se administró el 17 de enero del 2021 y, tras más de 190 millones de dosis administradas, el país registró una reducción de más del 50% en el número de casos y muertes por la infección al final del año 2021^(14)^.

Sin embargo, debido a las diferentes dinámicas de la infección, las tasas de vacunación, la aparición de variantes virales, el uso de medidas no farmacológicas y la planificación de los servicios de salud, el uso de EPI adecuados sigue siendo una condición esencial para la protección de los profesionales de salud, especialmente de los enfermeros^(15)^, contra COVID-19.

Un estudio realizado en Birjand, Irán, mostró que la baja seroprevalencia del SARS-CoV-2 entre los profesionales de salud puede explicarse por la buena adherencia a las medidas de prevención y control de infecciones y por el uso apropiado de EPI entre los profesionales^(15)^. Además, una revisión sistemática mostró que el uso de EPI confería una protección significativa contra la infección por COVID-19 en comparación con no usar EPI adecuado^(16)^.

Durante la pandemia de COVID-19, se reportaron estudios relacionados con los EPI para profesionales de salud como una forma de protección contra la enfermedad, pero la gran mayoría de las evidencias disponibles se centró en la disponibilidad y accesibilidad de los equipos^(17-18)^, la seguridad y eficacia^(19)^ y los efectos del uso prolongado^(20)^.

Por lo tanto, en virtud de la dinámica de la infección, de la vacunación y de la limitación de las investigaciones sobre el uso combinado de EPI en los períodos pre- y posvacunación contra la COVID-19 entre enfermeros —lo que culmina en un desconocimiento sobre el uso de los EPI recomendado por las agencias gubernamentales en diferentes períodos de la pandemia—, este estudio tiene como objetivo comparar el uso de EPI entre enfermeros en los períodos pre- y posvacunación contra COVID-19 en el estado de Paraíba.

## Método

### Diseño del estudio

Estudio transversal, analítico, basado en la herramienta *Strengthening the Reporting of Observational Studies in Epidemiology* (STROBE)^(21)^.

### Lugar y período

Este estudio se realizó en el estado de Paraíba, ubicado en la región Nordeste de Brasil. La encuesta se dividió en dos periodos distintos: el primero, entre el 1 de octubre y el 31 de diciembre del 2020; y el segundo, del 1 de noviembre del 2022 al 1 de mayo del 2023. Estos periodos corresponden a las etapas prevacunal y posvacunal contra COVID-19, respectivamente.

Con una población estimada de 3.974.687 habitantes, el primer periodo de la investigación arrojó una incidencia anual de 1.228,93 casos por cada 100.000 habitantes. La tasa de letalidad fue del 1,59%, mientras que la tasa de mortalidad fue de 19,51 muertes por cada 100.000 habitantes, lo que representa un total de 788 muertes registradas.

En la segunda etapa de la investigación, la tasa de letalidad fue del 0,27%, un total de 139 muertes registradas. No se encontraron datos específicos sobre la tasa de mortalidad durante este período.

### Población

Enfermeros del estado de Paraíba que actuaron en servicios públicos, privados o filantrópicos.

### Criterios de selección

Se consideraron elegibles los enfermeros que prestaron asistencia a personas en servicios públicos, privados o filantrópicos durante el período de pandemia de COVID-19 y que tenían acceso a internet. Se excluyeron aquellos que habían trabajado menos de seis meses después de la declaración de la pandemia de COVID-19.

### Definición de la muestra

Para la composición de la primera muestra, se consideraron 13.581 enfermeros de Paraíba; y para el segundo momento, 16.624, correspondientes al número total de enfermeros de Paraíba en el 2020 y el 2022^(22)^. Para el cálculo de la muestra, en ambos períodos se asumió un intervalo de confianza del 95%, un margen de error deseable del 5% y una frecuencia estimada del 50%. Luego se determinó una muestra de 375 y 377 enfermeros para el primer y el segundo períodos, respectivamente.

### Instrumento de recopilación de datos y variables del estudio

El instrumento de recopilación de datos para la primera etapa de la investigación se dividió en secciones con preguntas de opción múltiple. La primera sección incluyó datos sobre información sociodemográfica (sexo biológico, color/raza autodeclarado, estado civil y educación) y ocupacional (sector de actividad, tipo de institución en la que trabaja, asistencia en un hospital de campaña para COVID-19, tiempo de experiencia en la función); la segunda incluyó cuestiones relacionadas con el desempeño profesional y el contexto de COVID-19 (prestó asistencia en un hospital de campaña para COVID-19, diagnóstico de COVID-19, recibió capacitación o curso sobre la infección); y la tercera incluyó cuestiones sobre la disponibilidad y el uso de EPI (disposición por parte de la institución de trabajo de EPI suficientes y de calidad para su uso, los cuales se utilizan en la asistencia y en procedimientos generadores de aerosoles a personas con sospecha o con diagnóstico de COVID-19). El instrumento para la segunda etapa de recopilación de datos fue similar, excepto que se añadió una pregunta relacionada con el estado de vacunación (recibió la vacuna contra COVID-19).

El instrumento de recopilación de datos fue enviado a 15 evaluadores para fines de validación. Estos expertos completaron un formulario que contenía ítems para la evaluación general del instrumento de recopilación de datos (adecuación y aplicabilidad), de coherencia y adecuación a los objetivos de la investigación, así como ítems para evaluar la precisión científica y el contenido del instrumento e ítems para evaluar el lenguaje (adecuación, claridad, objetividad). Se utilizó el Índice de Validez de Contenido (IVC) y presentó valores superiores a 0,85, considerados válidos.

Se consideraron como variables dependientes el uso combinado de EPI en la asistencia directa y al realizar procedimientos que generen aerosoles al atender a personas con sospecha o con diagnóstico de COVID-19 (gorro, guantes, mascarillas N95, bata y pantalla facial/protector facial) en ambos momentos de la investigación.

### Recopilación de datos

Se invitó y reclutó en línea a enfermeros de Paraíba, utilizando el método *respondent-driven sampling* (RDS)^(23)^, adaptado para el entorno virtual. En este método, el enfermero participante reclutaba a otras personas que cumplían los mismos criterios de inclusión para participar en el estudio.

Cabe destacar que, para una mejor operativización de la recopilación de datos en línea, inicialmente se invitó a los investigadores a actuar en esta etapa de la investigación como reclutadores/recopiladores de participantes que cumplieran con los criterios de la investigación.

La implementación del RDS se inició con las coordinadoras de la investigación, quienes enviaron el cuestionario a sus pares profesionales, estudiantes de posgrado y exalumnos de grado. Los primeros participantes, llamados “semillas”, recibieron dos cupones de compensación social, que fueron distribuidos a otras dos personas, dando secuencia al proceso de reclutamiento en cadena. Cabe resaltar que los participantes no recibieron ningún incentivo financiero para participar en la investigación o para reclutar a nuevos participantes. Esta limitación se produjo debido a la falta de recursos financieros suficientes para cubrir todos los costos que implica el proceso de muestreo del RDS.

El equipo de recopiladores estuvo integrado por estudiantes de grado y posgrado y por profesionales de salud. Antes del reclutamiento, el equipo responsable realizó una capacitación preliminar mediante la plataforma *Google Meet*, en la cual se presentaron las propuestas y objetivos de la investigación, los criterios de elegibilidad, el método de reclutamiento de los participantes y el instrumento de recopilación de datos, con el fin de eliminar cualquier duda acerca del llenado y de la etapa de la investigación.

A continuación, los enfermeros que cumplieron con los criterios de inclusión fueron invitados a participar en la investigación mediante invitaciones enviadas por medio de las redes sociales (*WhatsApp*, *Instagram*, *Facebook*, correo electrónico, *LinkedIn*). Tras aceptar participar, se enviaba un enlace con información sobre la investigación y su confidencialidad. El enfermero, al hacer clic en el enlace de la investigación, era dirigido a la plataforma *SurveyMonkey*^®^ (primer momento) y *Research Electronic Data Capture* (*REDCap*^®^) (segundo momento), que permitía un solo envío del formulario por protocolo de internet (IP).

En la primera página del enlace, el participante accedía al Consentimiento Libre, Previo e Informado (CLPI) en dos copias, mediante su disponibilidad para descarga. Tras leer y confirmar su participación, mediante la opción “He leído y acepto participar en esta investigación”, el participante era dirigido al instrumento de recopilación de datos. Si no aceptaba participar, era dirigido a una página de cierre con un agradecimiento. El llenado del cuestionario llevaba entre cinco y siete minutos y el reclutador responsable esperaba el *feedback* en línea del participante con la confirmación de la finalización de la encuesta y para aclarar cualquier duda que pudiera surgir al responder el cuestionario.

### Tratamiento y análisis de datos

Los datos se exportaron a una hoja de cálculo de *Microsoft Excel* 2019 y, posteriormente, se importaron y se analizaron en el *software* estadístico R, versión 4.0.4, y en el *Statistical Package for the Social Sciences* (SPSS), versión 20. El análisis descriptivo se realizó utilizando la distribución de frecuencias absolutas y relativas para caracterizar la muestra.

Se realizaron análisis descriptivos con frecuencias absolutas y relativas. Para los análisis comparativos de las variables relacionadas con el uso combinado de EPI en la asistencia directa y en procedimientos generadores de aerosoles en personas con sospecha o con diagnóstico de COVID-19, se utilizó la prueba Z de proporciones^(24)^.

### Aspectos éticos

La investigación siguió las normas establecidas por la Resolución n.° 466/2012 del Consejo Nacional de Salud y las directrices para procedimientos en investigaciones en cualquier etapa en un entorno virtual, publicadas y comunicadas por la Comisión Nacional de Ética e Investigación (CONEP), el 3 de marzo del 2021, mediante la Circular n.º 02/2021^(25)^.

Este estudio está vinculado a dos proyectos de investigación, los cuales fueron aprobados por el Comité de Ética en Investigación (CEP) de la institución responsable, bajo el dictamen n.º 4.258.366/2020 (de 4 de septiembre del 2020) y n.° 5.542.659/2021 (de 25 de julio del 2022), respectivamente.

## Resultados

Inicialmente participaron en el estudio 579 enfermeros del estado de Paraíba. La mayoría era mujer (sexo asignado al nacer), 519 (89,6%); casada o en unión estable, 316 (54,6%); raza autodeclarada parda, 291 (50,3%); con títulos de posgrado, 424 (73,2%). En cuanto a la naturaleza de la institución de trabajo y al sector, la mayoría trabaja en instituciones públicas, 549 (94,8%); y en Unidades Básicas de Salud (UBS), 194 (33,5%), respectivamente (Tabla 1).

**Tabla 1-:** Características sociodemográficas, ocupacionales y relacionadas con COVID-19 de enfermeros en Paraíba (n = 579). Paraíba, Brasil, 2020

**Variables**	**n**	**%**
**Sexo**		
Femenino	519	89,6
Masculino	60	10,4
**Color/raza**		
Blanca	257	44,4
Parda	291	50,3
Negra	25	4,3
Amarilla	6	1
**Estado civil**		
Casado/unión estable	316	54,6
Soltero/divorciado	259	44,7
Viudo	4	0,7
**Escolaridad**		
Educación superior	155	26,8
Estudios de posgrado	424	73,2
Sector de actuación*		
Clínica ambulatoria	39	6,7
UCI ^†^	124	21,4
Enfermería	129	22,3
Centro quirúrgico	41	7,1
Unidad de Atención de Urgencias	59	10,2
Unidad de Emergencia	103	17,8
Unidad Básica de Salud	194	33,5
Consultorio privado	3	0,5
Otro	71	12,3
**Trabaja en una institución pública**		
Sí	549	94,8
No	30	5,2
**Trabaja en una institución privada**		
Sí	49	8,5
No	530	91,5
**Trabaja en una institución filantrópica**		
Sí	6	1
No	573	99
**Prestó asistencia en un hospital de campaña**		
Sí	157	27,1
No	422	72,9
**Le diagnosticaron COVID-19**		
Sí	174	30,1
No	405	69,9
**Recibió un curso o capacitación sobre COVID-19**		
Sí	387	66,8
No	192	33,2

*Más de una opción de respuesta; ^†^UCI = Unidad de Cuidados Intensivos

En el segundo período participaron en el estudio 734 enfermeros de Paraíba. La mayoría era mujer (sexo asignado al nacer), 642 (87,5%); casada o en unión estable, 372 (50,7%); raza autodeclarada parda, 388 (52,9%); con especialización o residencia, 440 (59,9%). En cuanto a la naturaleza de la institución donde trabajan, la mayoría trabaja en instituciones públicas, 622 (84,5%); y en el sector de trabajo departamentos de enfermería, 236 (32,2%) (Tabla 2).

**Tabla 2-:** Características sociodemográficas, ocupacionales y relacionadas con COVID-19 de los enfermeros de Paraíba (n = 734). Paraíba, Brasil, 2022-2023

**Variables**	**n**	**%**
**Sexo**		
Femenino	642	87,5
Masculino	92	12,5
**Color/raza**		
Blanca	284	38,7
Parda	388	52,9
Negra	52	7,1
Amarilla	8	1,1
Indígena	2	0,3
**Estado civil**		
Casado/unión estable	372	50,7
Soltero/divorciado	358	48,8
Viudo	4	0,5
**Escolaridad**		
Educación superior	193	26,3
Estudios de posgrado	541	73,7
Sector de actuación*		
Clínica ambulatoria	77	10,5
UCI ^†^	159	21,6
Enfermería	236	32,2
Centro quirúrgico	71	9,7
Unidad de Atención de Urgencias	127	17,3
Unidad de Emergencia	149	20,3
Unidad Básica de Salud	174	23,7
Consultorio privado	20	2,7
Otro	142	19,3
**Trabaja en una institución pública**		
Sí	622	84,5
No	112	15,5
**Trabaja en una institución privada**		
Sí	176	24
No	558	76
**Trabaja en una institución filantrópica**		
Sí	21	2,9
No	713	97,1
**Le diagnosticaron COVID-19**		
Sí	522	71,1
No	212	28,9
**Vacunado contra COVID-19**		
Sí	656	89,4
No	78	10,6
**Recibió un curso o capacitación sobre COVID-19 en el 2022**		
Sí	65	8,9
No	669	91,1

*Más de una opción de respuesta; ^†^UCI = Unidad de Cuidados Intensivos

La Tabla 3 muestra la frecuencia de uso de EPI en la asistencia directa y en procedimientos que generan aerosoles en personas con sospecha o con diagnóstico de COVID-19 en el 2020. En la asistencia directa, se observa que los EPI más utilizados fueron los guantes, seguidos de la pantalla facial, gorro, mascarilla N95 y bata impermeable. En cambio, en procedimientos generadores de aerosoles, el EPI más utilizado fue la mascarilla N95, seguido por la pantalla facial, guantes, gorro y bata impermeable.

La Tabla 4 muestra la frecuencia de uso de los EPI en la asistencia directa y en procedimientos generadores de aerosoles en personas con sospecha o con diagnóstico de COVID-19 en el período 2022-2023. Se observa que el EPI más utilizado fueron los guantes, seguido de la mascarilla N95, gorro, bata impermeable y pantalla facial. En cambio, en procedimientos generadores de aerosoles, el más utilizado fue la mascarilla N95, seguido de guantes, gorro, bata impermeable y pantalla facial.

Respecto a los resultados comparativos, la Tabla 5 muestra que existieron diferencias estadísticamente significativas entre los periodos pre- y posvacunación en el uso combinado de EPI en la asistencia directa y en procedimientos generadores de aerosoles en personas con sospecha o con diagnóstico de COVID-19. Se observa que, en el 2020, el porcentaje de uso combinado de EPI en la asistencia directa a personas con sospecha o con diagnóstico de COVID-19 por enfermeros de Paraíba fue mayor (50,6%), en comparación con el uso en el período 2022-2023 (34,5%) (p<0,01). Respecto al uso combinado de EPI en procedimientos generadores de aerosoles, también se observa que el porcentaje de uso fue mayor en el 2020 (49,7%), en comparación con el uso en el período 2022-2023 (32,1%) (p<0,01).

**Tabla 3-:** Distribución de los EPI utilizados por los enfermeros en el 2020 (n = 579). Paraíba, Brasil, 2020

EPI*	Asistencia directa a personas con sospecha o con diagnóstico de COVID-19 N (%)	Procedimientos generadores de aerosoles en personas con sospecha o con diagnóstico de COVID-19 N (%)
**Guantes**		
Sí	525 (90,7)	453 (78,2)
No	54 (9,3)	126 (21,8)
**Pantalla facial**		
Sí	508 (87,7)	479 (82,7)
No	71 (12,3)	100 (17,3)
**Gorro**		
Sí	503 (86,9)	444 (76,7)
No	76 (13,1)	135 (23,3)
**Mascarilla N95**		
Sí	477 (82,4)	491 (84,8)
No	102 (17,6)	88 (15,2)
**Bata impermeable**		
Sí	377 (65,1)	359 (62)
No	202 (34,9)	220 (38)

*EPI = Equipo de protección individual

**Tabla 4-:** Distribución de los EPI utilizados por los enfermeros en el período 2022-2023 (n = 734). Paraíba, Brasil, 2022-2023

EPI*	Asistencia directa a personas con sospecha o con diagnóstico de COVID-19 N (%)	Procedimientos generadores de aerosoles en personas con sospecha o con diagnóstico de COVID-19 N (%)
**Guantes**		
Sí	578 (78,7)	531 (72,3)
No	156 (21,3)	203 (27,7)
**Pantalla facial**		
Sí	368 (50,1)	399 (54,4)
No	366 (49,9)	335 (45,6)
**Gorro**		
Sí	512 (69,8)	457 (62,3)
No	222 (30,2)	277 (37,7)
**Mascarilla N95**		
Sí	544 (74,1)	581 (79,2)
No	190 (25,9)	153 (20,8)
**Bata impermeable**		
Sí	431 (58,7)	424 (57,8)
No	303 (41,3)	310 (42,2)

*EPI = Equipo de protección individual

**Tabla 5-:** Prueba de comparación de proporciones de uso combinado de EPI en la asistencia a personas con sospecha o con diagnóstico de COVID-19 entre enfermeros de Paraíba. Paraíba, Brasil, 2020/2022-2023

Uso combinado de EPI* en la asistencia directa			
EPI*	**% en el 2020**	**% 2022-2023**	**P-valor**
N95 + Guantes + Gorro + Bata + Pantalla facial	50,6	34,5	< 0,01 ^†^
Uso combinado de EPI* en procedimientos que generan aerosoles			
EPI*	**% en el 2020**	**% 2022-2023**	**P-valor**
N95 + Guantes + Gorro + Bata + Pantalla facial	49,7	32,1	**< 0,01†**

*EPI = Equipo de protección individual; ^†^p<0,05

## Discusión

Este estudio proporciona un análisis comparativo del uso combinado de EPI en la asistencia directa y en procedimientos generadores de aerosoles en personas con sospecha o con diagnóstico de COVID-19 entre enfermeros en el período pre- y posvacunación contra la infección. Debido a la limitación de investigaciones sobre el uso de EPI entre enfermeros en diferentes momentos de la pandemia, se utilizaron estudios realizados en períodos similares y con poblaciones similares con fines comparativos. De esta forma, la investigación aporta datos representativos de la región Nordeste de Brasil.

La mayoría de los encuestados en el estado de Paraíba es mujer de nacimiento, casada o en unión estable y tiene título de posgrado. Se sabe que la Enfermería es el mayor grupo ocupacional en el sector de salud, con alrededor de 27 millones de profesionales en todo el mundo^(26)^. La investigación de Enfermería en Brasil mostró que la mayoría es mujer (85,1%), el 48,7% está casada o en unión estable y el 41,5% es de raza parda^(27)^, datos que corroboran los hallazgos del estudio.

Se sabe que el uso de EPI es esencial para la protección de los profesionales de salud, así como de los pacientes. La literatura pone de manifiesto la importancia y eficacia del uso de EPI en el cuidado de personas con COVID-19, con la consecuente reducción de la contaminación^(7,28)^. En el estudio, se observa que los EPI más utilizados por los enfermeros en la asistencia, en ambos períodos, fueron los guantes, la mascarilla N95, el gorro, la bata impermeable y la pantalla facial, es decir, los enfermeros de Paraíba utilizaron al menos uno de estos EPI en el cuidado de la persona con sospecha o con diagnóstico de la infección. Datos que corroboran estudios realizados con profesionales de la salud en los estados de Ceará^(29)^ y Pernambuco^(30)^.

Los guantes, gorros y batas impermeables son EPI utilizados rutinariamente en la asistencia médica del paciente que sirven como barrera para que el patógeno no llegue a las superficies corporales, como las manos, el cabello y el cuerpo^(31-32)^. Respecto a COVID-19, revisiones sistemáticas han demostrado la eficacia de los EPI en la prevención de la infección, destacando que su protección se vuelve aún más efectiva cuando se combina con una adecuada higiene de manos, tanto antes como después de utilizar los equipos^(20,33)^.

Debido a la transmisión del SARS-CoV-2 por aerosoles, la mascarilla N95 y la pantalla facial se han convertido en EPI esenciales para el personal de enfermería, especialmente en procedimientos que generan aerosoles. Las investigaciones muestran que la N95, gracias a su capacidad de filtrar el 95% de las partículas aerosolizadas, ofrece una protección más efectiva contra los coronavirus que las mascarillas quirúrgicas^(34)^. La pantalla facial (*face shield*) proporciona protección de barrera para la superficie facial, lo que evita que gotículas, fluidos o aerosoles lleguen a la zona de los ojos, boca o nariz^(35)^. Cabe mencionar que los protectores faciales no sustituyen el uso de mascarillas, sino que actúan como coadyuvantes en la protección^(36)^.

En este estudio, se observa que la mascarilla N95 y la pantalla facial fueron los EPI utilizados con mayor frecuencia. Datos que corroboran un estudio realizado con 744 enfermeros de diferentes continentes^(37)^ y otro realizado en el estado de Pernambuco, Brasil^(30)^. Así pues, se constata que la mayoría de los enfermeros del estudio utilizaba los principales EPI, aunque fuera de forma individual.

Para una mayor protección del profesional de salud contra COVID-19, se recomendaron protocolos y directrices para adoptar el uso combinado de los principales EPI: batas, guantes, gorros, mascarilla quirúrgica o N95 y protección ocular (gafas protectoras o pantalla facial)^(8,38)^. En el 2020, los resultados mostraron que el 50,6% de los enfermeros combinó el uso de estos EPI en la asistencia directa de personas con sospecha o con diagnóstico de COVID-19. Resultado superior en comparación con un estudio realizado en Egipto, en la primera ola de COVID-19, en el que el 53,2% de los profesionales de salud, incluidos enfermeros, autoinformó que no utilizaba EPI^(39)^ y tuvo resultado inferior en comparación con un estudio realizado en Alemania, en el que la adhesión al uso combinado fue del 85%^(40)^.

Respecto al uso combinado de EPI en procedimientos generadores de aerosoles, el 49,7% de los enfermeros reportó su uso combinado. Estos datos son inferiores en comparación con los resultados de un estudio realizado con enfermeros en Arabia Saudita, donde el 100% utilizaba los EPI recomendados en procedimientos generadores de aerosoles^(41)^. Cabe destacar que Brasil, al inicio de la pandemia, se enfrentó a una escasez y distribución inadecuada de EPI, así como a una falta de cursos o capacitación sobre la infección y las técnicas correctas de paramentación y desparamentación, lo que puede haber incidido en el bajo uso y adhesión a los equipos.

En el período de 2022-2023, tras el inicio de la vacunación contra COVID-19, el 34,5% de los enfermeros de Paraíba utilizó EPI de forma combinada en la asistencia directa. Resultado inferior si se compara con un estudio realizado en Australia, con 2.197 profesionales de salud, que mostró que el 88,6% utilizaba EPI^(42)^ y en Dinamarca, con una tasa de adhesión del 84,5%^(43)^. Respecto al uso combinado en procedimientos generadores de aerosoles en personas con sospecha o con diagnóstico de COVID-19, el 32,1% de los enfermeros autoinformó su uso. Porcentaje bajo en comparación con un estudio realizado en países de ingresos medios y bajos, en el que el 49% y el 67% de los profesionales de la salud reportaron el uso combinado de EPI, respectivamente^(17)^.

Cabe señalar que el mundo vive un escenario diferente tras la vacunación masiva de la población contra COVID-19. Además, se ha suspendido el uso obligatorio de mascarillas en los servicios sanitarios, salvo en determinadas situaciones. El uso de EPI también puede verse influenciado por factores regionales, de disponibilidad, oferta de capacitación y miedo a la infección^(44)^. Estos factores pueden haber influido en el uso de EPI en la actualidad, lo que requiere una mirada crítica-reflexiva sobre la adhesión a las precauciones estándar en entornos sanitarios.

En el análisis comparativo, por medio de resultados estadísticamente significativos, el estudio mostró que en el año 2020 el porcentaje de uso de EPI por parte de los enfermeros en la asistencia directa (50,6%) fue mayor en comparación con el período 2022-2023 (34,5%). Se observó un resultado similar para el uso combinado en procedimientos generadores de aerosoles, en el que el 49,7% de los enfermeros los utilizó en el 2020 y el 32,1% en el período 2022-2023. Una disminución de aproximadamente un 17% para ambos.

Se trata de un resultado preocupante, dado que, a pesar de la disminución del número de casos y muertes, el SARS-CoV-2 sigue en circulación, e incluso después de una grave crisis sanitaria mundial causada por una enfermedad respiratoria, la adopción de equipos de protección individual en los servicios de salud aún puede estar descuidada. Un estudio realizado con profesionales de salud de Michigan, tras el inicio de la vacunación, mostró un aumento significativo en los sentimientos de seguridad tras la implementación de la vacuna, y la tasa de uso de mascarillas aumentó en el 2021^(45)^.

Además, los estudios muestran que la seguridad de las vacunas, el fin de la emergencia de salud pública, el agotamiento profesional debido al uso prolongado, la disminución de la percepción del riesgo y las creencias, sentimientos y barreras son factores que pueden incidir en el comportamiento preventivo y, en consecuencia, en el uso de EPI^(46)^. También es importante abordar que las creencias culturales de que el uso de EPI ya no es necesario y la menor percepción de riesgo frente al virus también pueden ser factores asociados a la disminución del uso.

Por lo tanto, el estudio aporta importantes aportes respecto a la necesidad de prácticas y políticas de salud dirigidas a la producción y gestión del cuidado en salud y de enfermería, especialmente en períodos de pandemia y relacionadas con el uso de EPI. Asimismo, enfatiza la necesidad de capacitación y educación sobre el uso adecuado de los EPI.

En cuanto a las limitaciones del estudio, se resalta un posible sesgo de selección, resultante de la mayor representatividad de profesionales de las UBS en el período anterior a la vacunación y de sectores de enfermería en el período posterior a la vacunación. Además, la recopilación de datos se produjo significativamente después de la implementación de la vacuna, lo que puede haber influido en los resultados.

## Conclusión

Los guantes, la pantalla o protector facial, el gorro, la mascarilla N95 y la bata impermeable fueron los EPI utilizados con más frecuencia por los enfermeros de Paraíba en la asistencia directa y en procedimientos generadores de aerosoles en personas con sospecha o con diagnóstico de COVID-19. Al comparar el uso de EPI en el período pre- y posvacunación contra la COVID-19, se observó que el uso combinado de EPI fue porcentualmente mayor en el 2020, es decir, en el período anterior a la vacunación contra COVID-19, en comparación con el uso en el período 2022-2023.

Por lo tanto, sigue siendo necesario fortalecer la educación permanente en las instituciones de salud frente a los nuevos escenarios y comportamiento de la COVID-19, ofreciendo capacitaciones sobre la paramentación adecuada, así como actualizar la adhesión a las recomendaciones técnicas o protocolos sobre el uso de EPI como barrera de protección respiratoria y el uso adecuado de estos equipos. Garantizar la seguridad y el bienestar del personal de enfermería es esencial para combatir las pandemias de enfermedades respiratorias.

## Data Availability

Los conjuntos de datos relacionados con este artículo estarán disponibles previa solicitud al autor correspondiente.
